# New Invariant Expressions in Chemical Kinetics

**DOI:** 10.3390/e22030373

**Published:** 2020-03-24

**Authors:** Gregory S. Yablonsky, Daniel Branco, Guy B. Marin, Denis Constales

**Affiliations:** 1 McKelvey School of Engineering, Department of Energy, Environmental and Chemical Engineering, Washington University in St. Louis, 1 Brookings Dr., St. Louis, MO 63130, USA; 2 Department of Separation and Conversion Technology, VITO (Flemish Institute for Technological Research), Boeretang 200, B-2400 Mol, Belgium; Daniel.BrancoPinto@vito.be; 3 Laboratory for Chemical Technology, Ghent University, Technologiepark 914, B-9052 Ghent, Belgium; Guy.Marin@UGent.be; 4 Department of Electronics and Information Systems ELIS, Ghent University, Building S-8, Krijgslaan 281, B-9000 Ghent, Belgium; Denis.Constales@UGent.be

**Keywords:** invariant expression, two-step mechanism, scaled incremental conversion, conservatively perturbed equilibrium, linear complex mechanism, thermodynamic invariant

## Abstract

This paper presents a review of our original results obtained during the last decade. These results have been found theoretically for classical mass-action-law models of chemical kinetics and justified experimentally. In contrast with the traditional invariances, they relate to a special battery of kinetic experiments, not a single experiment. Two types of invariances are distinguished and described in detail: thermodynamic invariants, i.e., special combinations of kinetic dependences that yield the equilibrium constants, or simple functions of the equilibrium constants; and “mixed” kinetico-thermodynamic invariances, functions both of equilibrium constants and non-thermodynamic ratios of kinetic coefficients.

## 1. Introduction

### 1.1. Definition of Invariants

Searching for invariants is one of the most important goals of many sciences such as chemical kinetics and chemical engineering. Invariants are considered functions of the state variables that remain constant during non-steady-state complex transformations.

Linear element conservation laws are well known linear invariances that are widely used in chemistry and chemical engineering. Linear stoichiometric relationships of chemical reactions are cases of conservation laws. Stoichiometric coefficients are numbers of molecules of chemical components, which participate in chemical reactions. These coefficients have a negative sign and a positive sign for reactants and products, respectively.

Linear element conservation laws are valid regardless of the kinetic and thermodynamic properties of the reaction mechanism, as well as the way the chemical reactions are carried out. These laws are determined only by the list of chemical substances. As for linear stoichiometric relationships, they typically correspond to the detailed mechanism of a complex chemical reaction. The up-to-date mathematical framework of application of these linear invariants is presented in recent monographs [1,2].

### 1.2. Thermodynamic Invariants from Reciprocal Experiments

Since 2011 new and original types of chemical invariants were described [3,4,5]. These invariants of thermodynamic origin are closely related to Onsager’s famous reciprocal relations [6,7]. The experimental procedure, real or computational, consists of two dual experiments performed from different initial conditions of the reacting mixture, called the “dual experiments”. The simplest of these invariants is related to the single reversible reaction A ⇄ B, in a batch reactor (BR):

- The first experiment is performed in a reactor primed with substance A only.
- The second experiment is performed in a reactor primed with substance B only.

In both cases, the time-dependent concentrations of A and B are measured, A(t) and B(t), respectively. A special attention was paid to symmetric concentration profiles: the dependences “B produced from pure A”, B_A_(t), from the first experiment, and “A produced from pure B”, A_B_(t), from the second experiment. Explicit formulas for these concentration profiles are shown in Table 1, assuming that both the forward and backward reaction as first-order, monomolecular reactions, with kinetic coefficients k^+^ and k^−^, respectively.

**Figure 1:**
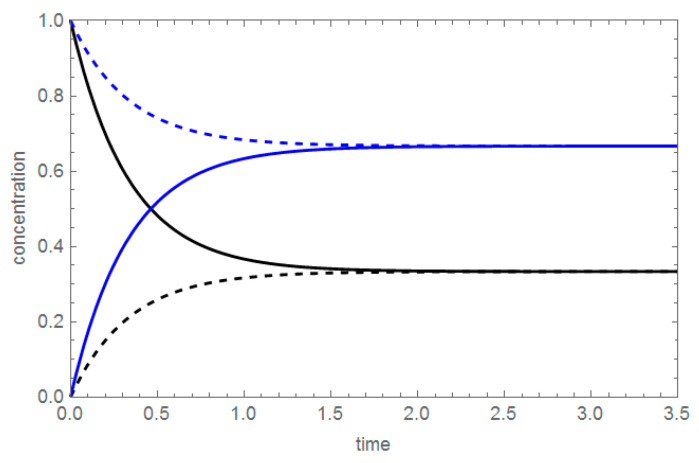
Concentration profiles of A (black) and B (blue), starting from pure A (solid) and from pure B (dashed), assuming a single step reversible reaction A ⇄ B with k^+^= 2 s^−1^, k^−^= 1 s^−1^. The ratio between B_A_(t) (solid blue) and A_B_(t) (black dashed) is the equilibrium constant.

The notation of the concentration profiles is as follows: the first capital letter denotes the substance, whereas the subscript letter denotes the single component primed in the reactor, in this case: pure A or pure B. The concentration profiles shown in Table 1 are plotted in Figure 1.

As seen in Table 1, the ratio of the symmetric concentration profiles B_A_(t)/A_B_(t) is constant, equal to the equilibrium constant of the reversible reaction K_eq_, K_eq_ = k^+^/k^−^. The equality B_A_(t)/A_B_(t) = K_eq_ is valid for t > 0, i.e., throughout the course of the reaction. Clearly, this invariant expression is different from other linear invariances such as mass conservation balances. The new invariant expression is used as follows: knowing the thermodynamic characteristic—the equilibrium constant—and one concentration profile, say, A_B_(t), we can find another, unknown, concentration profile, for instance B_A_(t) = A_B_(t)K_eq_ [8].

This result is valid also for a steady-state plug flow reactor (PFR) and a steady-state continuously stirred tank reactor (CSTR), if the astronomic time t is replaced by the space time τ, defined as the reactor volume divided by the volumetric flow rate [1,2].

It is reasonable to define this ratio of concentration profiles, B_A_(t)/A_B_(t), as a thermodynamic invariant, since it is equal to a thermodynamic parameter such as the equilibrium constant K_eq_. This type of invariant can be observed in more complicated, reversible linear mechanisms, calculated from the ratio of concentration profiles of any arbitrary chemical species connected via any number of reversible reactions, as long as these concentration profiles are obtained from dual experiments [4]. The thermodynamic invariants obtained for complex multistep mechanisms are two-fold:

- Pure equilibrium constants, obtained from the ratio of concentration profiles of chemical species connected via a single step reaction within a complex chemical mechanism.
- Apparent equilibrium constants, consisting of products of equilibrium constants of elementary reactions, obtained from the ratio of concentration profiles of chemical species connected via multiple step reactions in a complex chemical mechanism.

The theoretical basis of these invariants within the thermodynamic theory of irreversible processes is given elsewhere [4]. In 1931, Onsager [6,7] presented the foundations and generalizations of the reciprocal relations introduced in the 19^th^ century by Lord Kelvin and Helmholtz. In his historical papers, Onsager mentioned also the close connection between these relations and the detailed balance of elementary processes: at equilibrium, each elementary transaction must be equilibrated by its inverse transaction. For linear or linearized kinetics with microreversibility, x’ = K x, where x is the vector of the solution, the kinetic operator K is symmetric in the entropic inner product. This form on Onsager’s reciprocal relations implies that the shift in time, e^Kt^, is also a symmetric operator. This feature generates the reciprocal relations between the kinetic curves; this is the fundamental basis of our thermodynamic invariants [4].

In a more general setting, duality between experiments must be defined using the entropic inner product [4]. Let J denote the vector of fluxes and X that of thermodynamic forces, then by Onsager’s relations J = L X, where L is a symmetric matrix. In isolated systems, X is the gradient of the entropy Φ, and the linear(ized) kinetic equation is $\dot{x}=\mathrm{Kx}$, where $K=L{\left(D^{2}\Phi \right)}_{\mathrm{eq}}$ is the product of two symmetric matrices, which need not be symmetric (for the standard inner product). If, however, we use the entropic inner product instead, defined by $<a|b{>}_{\Phi }=-\sum_{i,j}a_{i}\frac{\partial^{2}\Phi }{\partial x_{i}\partial x_{j}}{|}_{\mathrm{eq}}b_{j},\;$symmetry is obtained in the sense that $<\mathrm{Ka}\left|b{>}_{\Phi }=<a\right|\mathrm{Kb}{>}_{\Phi }$. Integrated over time, this means that $<e^{\mathrm{Kt}}a\left|b{>}_{\Phi }=<a\right|e^{\mathrm{Kt}}b{>}_{\Phi }.$ The general requirement for two trajectories to be dual is then that their initial values be orthogonal in this entropic inner product.

Even some simple non-linear mechanisms may show similar invariants, calculated from the ratio of selected concentration profiles. For instance, for the elementary reaction A + B ⇄ C + D it can be demonstrated that (C_A_(t) D_C_(t))/(A_C_(t) B_A_(t)) = K_eq_, where C_A_(t) and B_A_(t) are concentration profiles obtained when the initial concentration of C is zero, and A_C_(t) and D_C_(t) are concentration profiles obtained when the initial concentration of A is zero [5]. 

With this knowledge, it is possible to predict unknown kinetic dependences based on the chemical equilibrium description and known kinetic dependences [8]. Additionally, we are able to confirm our experimental data validating them via the new invariants. Design of special batteries of kinetic experiments, virtual and/or real, can be considered a new step towards understanding the behavior of complex chemical reactions, and to gain insights on the intrinsic kinetic features of complex mechanisms. 

## 2. Experimental Verification of Thermodynamic Invariants

### 2.1. Water Gas Shift Reaction 

Yablonsky et al. [4] and then Constales et al. [9] justified the experimental validity of these invariances for non-steady-state kinetic dependences in a Temporal Analysis of Products (TAP) reactor. The experiments were performed in the thin zone TAP reactor Knudsen regime conditions. The reversible water–gas shift reaction, H_2_O + CO ⇄ CO_2_ + H_2_, was carried over an iron oxide catalyst. Two types of pulse experiments were performed: a) the oxidized catalyst was treated by a series of pulses of CO, and b) the reduced catalyst was treated by a series of pulses of CO_2_. A single pulse experiment was performed injecting CO in the reactor, and measuring the exit flow of CO_2_, and vice versa: injecting CO_2_ in the reactor, and tracking the flow of CO at the exit of the reactor. The reversible conversion of CO to CO_2_ was approximated by a first order reversible reaction A ⇄ B. From the combined Laplace–Fourier technique, the thermodynamic invariant was obtained from the outlet flow data of the two gases.

### 2.2. Redox Reaction of Ferricyanide and Ferrocyanide

Later, Hankins et al. [10] performed transient electrochemical experiments using the reduction of ferricyanide to ferrocyanide, measuring the concentration of each substance separately using a gold disk-ring electrode. The electrochemical reaction ferricyanide ⇄ ferrocyanide was approximated by a first order reversible reaction A ⇄ B. The experiments consisted of a dual chronoamperometry, by setting the potential of both the ring and disk electrodes to an equivalent far-from-equilibrium potential such as the anodic or cathodic limit, respectively, and allowing relaxation to equilibrium state defined by the Nernst potential. The limiting electric currents are related by the equilibrium constant of the ferri/ferrocyanide system. This relationship provides the unique possibility of predicting the transient electrochemical trajectory starting from one initial condition based only on both the known trajectory, which starts from the symmetric initial condition and the equilibrium constant.

### 2.3. Etherification/Hydrolysis Reaction

Peng et al. [11] tested the validity of the thermodynamic invariance using a batch reactor where the reaction of etherification of ethanol with acetic acid was studied jointly with the reaction of hydrolysis of ethyl acetate. In the etherification reaction, the glass flask was loaded with ethanol and acetic acid, and with ethyl acetate and water for the hydrolysis reaction, using acetonitrile as a solvent, at different temperatures (20, 30 and 40 °C); ethanol + acetic acid ⇄ ethyl acetate + water, or, symbolically, A + B ⇄ C + D, where A is ethanol, B is acetic acid, C is ethyl acetate and D is water. As mentioned in the previous section, this reaction has an invariant expression, (C_A_(t) D_C_(t))/(A_C_(t) B_A_(t)) = K_eq_. This was the first experimental evidence of a thermodynamic invariance obtained for a non-linear chemical system, and this invariance was found in the dual kinetic experiment [5,11].

## 3. Kinetico-Thermodynamic Invariants for Two-Step Mechanisms

The single reversible reaction A ⇄ B is the starting point of the thermodynamic invariants discussed in the previous section. In this section it will be shown that more complex linear mechanisms exhibit the so called kinetico-thermodynamic invariants. The two-step mechanism A ⇄ B ⇄ C is chosen for illustrating these invariants. Of the two methods described in this section to obtain kinetico-thermodynamic invariants, only the last one, described in Section 3.2, can be extrapolated to more complex linear mechanisms.

### 3.1. From Scaled Incremental Conversion (SCI)

The function that will be used to calculate the invariants for a two-step mechanism is closely related to the widely used term “conversion”. For the substance A, the Scaled Incremental Conversion (SIC) of A, χ_A_, is defined as follows:

(1) $$\chi_{A}=\frac{A\left(t\right)-A_{o}}{A_{\mathrm{eq}}-A_{o}}$$

where A_o_ and A_eq_ are the initial and the equilibrium concentration of A, respectively. 

**Figure 2:**
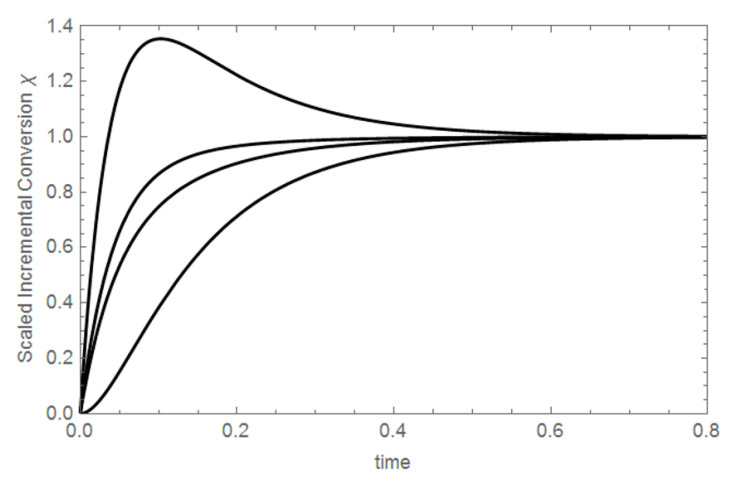
From top to bottom, the Scaled Incremental Conversion (SIC) plots of C_B_, B_B_, B_A_ and C_A_. k_1_^+^ = 4.5 s^−1^, k_1_^−^ = 10 s^−1^, k_2_^+^ = 6.5 s^−1^, k_2_^−^ = 12 s^−1^.

At the beginning of the reaction, at time t = 0, A(0) = A_o_, so the SIC value χ is zero. On the other hand, at the end of the reaction or at equilibrium, A(t→∞) = A_eq_, and the SIC value χ is equal to one, as seen in Figure 2. For both irreversible and reversible reactions, the SIC values go from zero to one, at the beginning of the chemical reaction and at chemical equilibrium, respectively. It can be easily demonstrated that SIC expressions of two chemical species from symmetric initial conditions are always equal [12].

The invariants are calculated using the invariant generator function F shown in Equation (2). This function uses four concentration profiles C_i_ as arguments:

(2) $$F\left(C_{1},C_{2},C_{3},C_{4}\right)=\frac{{\Delta \chi }_{12}}{{\Delta \chi }_{34}}=\frac{\chi_{1}-\chi_{2}}{\chi_{3}-\chi_{4}}$$

where χ_i_ denotes the SIC of the substance i, evaluated at a given initial condition. 

The difference of SIC terms that appear in the generator function F is equal to zero at t = 0, and is also zero at equilibrium. Then, a plot of the difference of SIC terms shows an extreme value at a value of time defined exclusively by the kinetic coefficients, independent of the SIC terms involved in the difference [12]. 

The invariants obtained from Equation (1) for a two-step consecutive mechanism will be divided threefold, according to the number of initial conditions involved in the four arguments of the invariant generator function F:

• Thermodynamic invariants, calculated from the same initial condition. These invariants, shown in Table 2, depend on two independent parameters: the equilibrium constants K_1_ and K_2_. 

To facilitate the reading of the tables of invariants, we will break down the last of the invariant expressions listed in Table 2. The arguments of this invariant are {A_B_, B_B_, B_B_, C_B_}; these concentration profiles are the four arguments of the function F. The invariant expression is obtained evaluating SIC expressions of these concentration profiles, and relating them according to Equation (2), as follows:

(3) $$\begin{array}{ll} F\left(A_{B},B_{B},B_{B},C_{B}\right) & =\frac{\chi_{A_{B}}-\chi_{B_{B}}}{\chi_{B_{B}}-\chi_{C_{B}}}=\frac{{\frac{A\left(t\right)-A_{o}}{A_{\mathrm{eq}}-A_{o}}|}_{\left(A_{o},B_{o},C_{o}\right)=\left(0,B_{o},0\right)}-{\frac{B\left(t\right)-B_{o}}{B_{\mathrm{eq}}-B_{o}}|}_{\left(A_{o},B_{o},C_{o}\right)=\left(0,B_{o},0\right)}}{{\frac{B\left(t\right)-B_{o}}{B_{\mathrm{eq}}-B_{o}}|}_{\left(A_{o},B_{o},C_{o}\right)=\left(0,B_{o},0\right)}-{\frac{C\left(t\right)-C_{o}}{C_{\mathrm{eq}}-C_{o}}|}_{\left(A_{o},B_{o},C_{o}\right)=\left(0,B_{o},0\right)}}\\ & =\frac{\frac{A_{B}\left(t\right)}{A_{\mathrm{eq}}}-\frac{B_{B}\left(t\right)-B_{o}}{B_{\mathrm{eq}}-B_{o}}}{\frac{B_{B}\left(t\right)-B_{o}}{B_{\mathrm{eq}}-B_{o}}-\frac{C_{B}\left(t\right)}{C_{\mathrm{eq}}}}=\frac{k_{1}^{+}}{k_{1}^{-}}\frac{k_{2}^{+}}{k_{2}^{-}}=K_{1}K_{2}=K_{12} \end{array}$$

Due to the fact that the SIC expressions of reciprocal concentration profiles are equal, this invariant expression can also be obtained from the following combinations of arguments:

(4) $$F\left(A_{B},B_{B},B_{B},C_{B}\right)=F\left(B_{A},B_{B},B_{B},C_{B}\right)=F\left(A_{B},B_{B},B_{B},B_{C}\right)=F\left(B_{A},B_{B},B_{B},B_{C}\right)=K_{12}$$

• Kinetico-thermodynamic invariants calculated from two different initial conditions, 

Since the two-step mechanism is described by four parameters: k_1_^+^, k_1_^−^, k_2_^+^ and k_2_^−^, the kinetico-thermodynamic invariants are functions of three independent dimensionless parameters: two equilibrium constants K_1_ = k_1_^+^/ k_1_^−^ and K_2_ = k_2_^+^/ k_2_^−^, and the non-thermodynamic ratio κ_in_ = k_1_^+^/k_2_^−^, as seen in Table 3 and Table 4. Any other ratio of kinetic coefficients can be calculated from both the equilibrium constants and κ_in_; however, it is not possible to resolve single kinetic coefficients using the invariants. The physicochemical meaning of $\kappa_{\mathrm{in}}$ is the following: it is the ratio of the kinetic coefficients of the incoming reactions to the substance B. A similar non-thermodynamic ratio is κ_out_ = k_1_^−^/k_2_^+^; the ratio of the kinetic coefficients of the outgoing reactions to the substance B. These non-thermodynamic ratios are related as follows:

(5) $$\frac{\kappa_{\mathrm{in}}}{\kappa_{\mathrm{out}}}=K_{1}K_{2}=K_{12}$$

• Kinetico-thermodynamic invariants calculated from three different initial conditions, shown in Table 5. 

### 3.2. From Conservatively Perturbed Equilibrium (CPE). The Simplest Case 

If two equilibrium concentrations are swapped in a two-step mechanism, the initial concentration of the third one will be equal to its corresponding equilibrium concentration, due to balance. This type of experiment is denoted as Conservatively Perturbed Equilibrium (CPE), described elsewhere [13]; in a CPE experiment, the initial concentrations of some substances are set equal to their corresponding equilibrium concentrations; these substances are called unperturbed substances. For the rest of the substances in the mechanism, called perturbed substances, their initial concentrations differ from their corresponding equilibrium concentrations, in such a way that the total balances of the elements remains unaffected. A special parameter, δ, measures the magnitude of the perturbation for these substances with respect to their corresponding equilibrium concentrations.

There are three different possibilities of CPE-experiments on the two-step mechanism studied in this section, shown in Table 6. Notice that the total balance of the perturbed and unperturbed substances is not affected by the perturbations. For instance, for the case when B and C are perturbed, and A unperturbed, we have that A_o_ + B_o_ + C_o_ = A_eq_ + (B_eq_ – δ) + (C_eq_ + δ) = A_eq_ + B_eq_ + C_eq_. When δ = 0, detailed balance is obtained. For a special value of δ, shown in the last column in Table 6, the equilibrium concentrations of the perturbed substances appear swapped. This value of δ corresponds to the absolute value of the difference of the equilibrium concentrations of the swapped substances. Then, the swap of the equilibrium concentrations it is a particular case of a CPE experiment [14].

**Figure 3:**
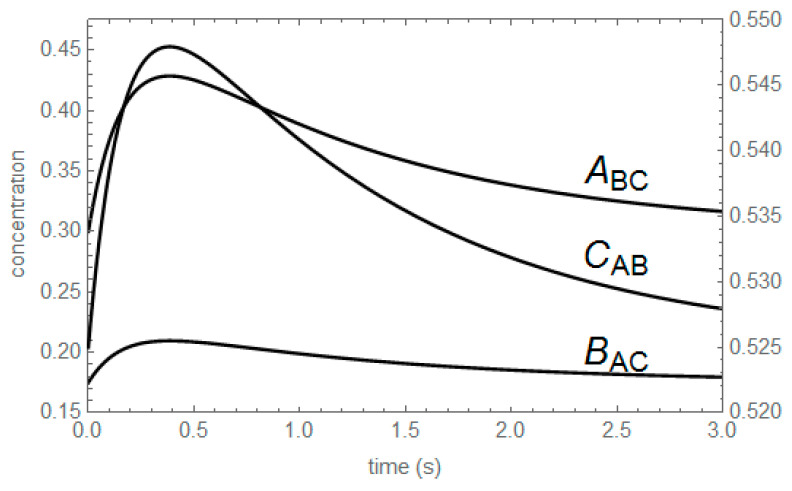
Concentration profiles of A_BC_, B_AC_ and C_AB_. The first two, A_BC_ and B_AC_, must be read with the left Y-scale. The last one, C_AB_, must be read with the right Y-scale. k_1_^+^ = 1.75 s^−1^, k_1_^−^ = 3.00 s^−1^, k_2_^+^ = 1.50 s^−1^, k_2_^−^ = 0.50 s^−1^.

**Figure 4:**
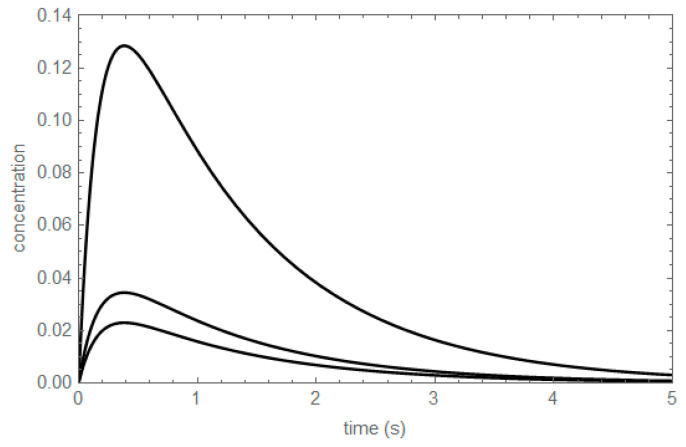
Corrected concentration profiles of A_BC_, B_AC_ and C_AB_. From top to bottom, A_BC_ − A_eq_, B_AC_ − B_eq_ and C_AB_ − C_eq_. k_1_^+^ = 1.75 s^−1^, k_1_^−^ = 3.00 s^−1^, k_2_^+^ = 1.50 s^−1^, k_2_^−^ = 0.50 s^−1^.

The concentration profiles of the unperturbed substances shown in Table 6 are shown in Figure 3: A_BC_, B_AC_ and C_AB_; the subscripts indicate the substances whose equilibrium concentrations are swapped. For instance, A_BC_ denotes the concentration profile of A, being A unperturbed, swapping the equilibrium concentrations of B and C, i.e., from the initial conditions (A_o_, B_o_, C_o_) = (A_eq_, B_eq_ – δ, C_eq_ + δ). The corrected concentration profiles shown in Figure 4 are proportional by simple functions of the kinetic coefficients, because the extreme values occur all at the same value of time.

There are only two independent ratios of corrected concentration profiles of unperturbed substances in the two-step mechanism studied:

(6) $$\{\begin{array}{c} \frac{A_{\mathrm{BC}}\left(t\right)-A_{\mathrm{eq}}}{B_{\mathrm{AC}}\left(t\right)-B_{\mathrm{eq}}}=\left(\frac{k_{1}^{-}}{k_{1}^{+}-k_{2}^{-}}\right)\frac{\delta_{1}}{\delta_{2}}=\left(\frac{\kappa_{\mathrm{in}}}{K_{1}\left(\kappa_{\mathrm{in}}-1\right)}\right)\frac{\delta_{1}}{\delta_{2}}\\ \frac{A_{\mathrm{BC}}\left(t\right)-A_{\mathrm{eq}}}{C_{\mathrm{AB}}\left(t\right)-C_{\mathrm{eq}}}=-\frac{k_{1}^{-}}{k_{2}^{+}}\frac{\delta_{1}}{\delta_{2}}=-\kappa_{\mathrm{out}}\frac{\delta_{1}}{\delta_{2}}\; \end{array}$$

The subscripts in δ_1_ and δ_2_ appear in the last equation to stress that the values of δ in the numerator and the denominator of the ratio can be different. Evaluation of the invariant expressions shown in Equation (6) yields the same time-independent functions of kinetic coefficients, regardless of the type of chemical reactor.

The values obtained from the invariant expressions shown in Equation (6) are shown in Table 7 and Table 8, for different values of δ_1_ and δ_2_. The values of δ_1_ are shown in the first row, whereas the values of δ_2_ are shown in the first column. The values of δ on the first two columns and the first two rows yield an initial concentration of zero for one of the substances of the two-step mechanism. The values of δ on the third column and the third row yield swapped equilibrium concentrations. As δ measures the difference between the initial concentrations and the corresponding equilibrium concentrations, it is convenient to choose its value as equilibrium concentrations, or differences of equilibrium concentration. 

It is possible to obtain invariant expressions for a two-step linear mechanism also mixing the two procedures just described, as the ratio of the difference of two SIC terms and a corrected concentration profile. The thermodynamic invariants obtained from this ratio are shown in Table 9. In the first column, it is shown the invariant expression and its value, and in the rest of the columns is shown the value of the invariant expression for different values of δ.

## 4. Kinetico-Thermodynamic Invariants for Linear Complex Mechanisms

### 4.1. Invariant Expressions for Polar Two-Step Mechanisms

In the two-step mechanism A ⇄ B ⇄ C, the single-step substances A and C participate in the single reactions A ⇄ B and B ⇄ C, respectively; these single-step substances can be considered as poles of the two-step mechanism. We will demonstrate that if a complex linear mechanism contains a polar two-step submechanism, the invariant expressions shown in the previous section for the two-step mechanism A ⇄ B ⇄ C, Equation (6), are also valid for the polar two-step submechanism. In other words, the invariant expressions remain unaltered if other parallel first-order reactions occur from the substance B, the single substance that connects the two poles A and C. In Table 10 are shown several linear mechanisms with one or more polar two-step submechanisms. 

An important requirement to be fulfilled is that the initial concentrations of the chemical species apart from the polar two-step submechanism must be equal to the equilibrium concentrations; i.e., they are unperturbed substances. The two perturbed substances belong to the polar two-step submechanism, and the third substance in this submechanism remains unperturbed. Corrected ratios of the concentration profiles of the unperturbed substances within the polar two-step submechanism yield the invariant expressions, the same expressions shown in Equation (6). A formal demonstration of this feature is shown elsewhere [15]. 

### 4.2. Invariant Expressions for Linear Mechanisms with Single-Step Substances

An invariant expression can be constructed for any linear complex mechanism with at least two single-step substances, similar to the last equation in Equation (6). These single-step substances can be connected by a single substance, like in a polar two-step submechanism, or by any linear complex submechanism. The invariant expression can be determined for whichever single-step substances within a linear complex mechanism. The value of the invariant expression will be a generalization of the last equation in Equation (6) [16].

Consider the following complex mechanism:

$$A_{1}\begin{array}{c} k_{1}^{+}\\ ⇄\\ k_{1}^{-} \end{array}A_{2}\begin{array}{c} k_{2}^{+}\\ ⇄\\ k_{2}^{-} \end{array}A_{3}\ldots A_{n-1}\begin{array}{c} k_{n-1}^{+}\\ ⇄\\ k_{n-1}^{-} \end{array}A_{n}$$

where A_i_ are chemical species, and k_i_^+^ and k_i_^−^ are the kinetic coefficients of the forward and backward i-th reaction, respectively. In this mechanism, there are two single-step substances, A_1_ and A_n_. Although not explicitly shown, other parallel reactions can occur from substances A_i_, 2 ≤ i ≤ n−1; if so, more than two single-step substances exist. These parallel reactions do not affect the invariant expression.

The formula for the invariant expression of the linear complex mechanism shown above is:

(7) $$\frac{A_{1\left(n-1,n\right)}-A_{1,\mathrm{eq}}}{A_{n\left(1,2\right)}-A_{n,\mathrm{eq}}}=-\frac{\prod_{i=1}^{n-2}k_{i}^{-}}{\prod_{i=2}^{n-1}k_{i}^{+}}\frac{\delta_{1}}{\delta_{2}}=\frac{k_{1}^{-}}{k_{n-1}^{+}}\left(\frac{1}{\prod_{i=2}^{n-2}K_{i}}\right)\frac{\delta_{1}}{\delta_{2}}$$

where A_i(j,k)_ is the concentration profile of A_i_, with the substances A_j_ and A_k_ as perturbed substances; the rest of the chemical species in the complex mechanism remain unperturbed. A_i_,_eq_ denotes the equilibrium concentration of the substance A_i_, and δ_1_ and δ_2_ are perturbations in the numerator and the denominator, respectively. In Table 11 are shown several linear mechanisms with one or more polar two-step submechanisms.

The non-thermodynamic ratio of kinetic coefficients k_1_^−^/k_n−1_^+^ that appears in the invariant expression shown in Equation (7) can be seen as the ratio of the kinetic coefficients of the outgoing reactions to the submechanism that connects the single-step substances A_1_ and A_n_. In a polar two-step submechanism A_1_ ⇄ A_2_ ⇄ A_3_, this non-thermodynamic ratio is of the kinetic coefficients of the outgoing reactions to the single substance A_2_; this ratio corresponds to κ_out_, defined in Section 1.2.

Summarizing, the invariant expression derived in this section, shown in Equation (7), consists of the product of three factors of different nature:

A non-thermodynamic factor: the ratio of kinetic coefficients of outgoing reactions, either to a single substance or to a submechanism of the complex mechanism.

A thermodynamic factor: the pure or apparent equilibrium constant of a submechanism of the complex mechanism. The inverse of this factor appears in the invariant expression. 

An “experimental” factor: the values of the perturbations δ_1_ and δ_2_, defined by the experimental setup of the two experiments needed to determine the invariant expression.

## 5. Final Remarks

Design of special batteries of kinetic experiments, virtual and/or real, can be considered a new step towards understanding the behavior of complex chemical reactions. It was found, analytically, computationally and experimentally, that some sets of kinetic experiments, e.g., reciprocal experiments with symmetric initial conditions, experiments under “conservatively perturbed equilibrium” conditions, exhibit invariances. Such invariant expressions are ratios of non-steady-state dependences carefully chosen that yield constants at any moment of time. These invariant expressions differ from the invariances previously known (mass conservation laws and stoichiometric relationships). Two groups of such invariances can be distinguished:

- Thermodynamic invariances as functions of equilibrium constants.
- “Mixed” kinetico-thermodynamic invariances as functions of both equilibrium constants and kinetic coefficients. These invariants were found for special linear mechanism: two-step mechanisms, and other linear mechanisms with polar two-step submechanisms and single-step substances.

A present dogma in chemical kinetics states that it is impossible to describe the non-equilibrium behavior of a chemical system based exclusively on its description in equilibrium, except for some linear relationships near the vicinity of equilibrium [3]. However, the research that led us to obtain the thermodynamic invariant expressions shows that some dependences that describe kinetic behavior, far from equilibrium, are related via a thermodynamic parameter such as the equilibrium constant. With this knowledge, it is possible to predict unknown kinetic dependences based on the chemical equilibrium description and known kinetic dependences. 

## Figures and Tables

**Table 1:** Concentration profiles of A and B for the reversible reaction A ⇄ B, from two different initial conditions.

Experiment	Substance	Concentration Profile
1st experiment, from pure A.	A	$A_{A}\left(t\right)=\frac{k^{+}e^{-\left(k^{+}+k^{-}\right)t}+k^{-}}{k^{+}+k^{-}}$
	B	$B_{A}\left(t\right)=\frac{k^{+}\left(1-e^{-\left(k^{+}+k^{-}\right)t}\right)}{k^{+}+k^{-}}$
2nd experiment, from pure B.	A	$A_{B}\left(t\right)=\frac{k^{-}\left(1-e^{-\left(k^{+}+k^{-}\right)t}\right)}{k^{+}+k^{-}}$
	B	$B_{B}\left(t\right)=\frac{k^{-}e^{-\left(k^{+}+k^{-}\right)t}+k^{+}}{k^{+}+k^{-}}$

**Table 2:** Thermodynamic invariants for a two-step consecutive mechanism, calculated from the same initial condition.

Arguments of the F function	Invariant
$\left\{A_{A},B_{A},A_{A},C_{A}\right\}$	$-K_{2}$
$\left\{A_{B},B_{B},B_{B},C_{B}\right\}$	$K_{12}$

**Table 3:** Kinetico-thermodynamic invariants for a two-step consecutive mechanism, calculated from two different initial conditions: A and B.

Arguments of the F function	Invariant
$\left\{A_{A},B_{A},B_{A},B_{B}\right\}$	$-\frac{\left(1+K_{12}\right)\kappa_{\mathrm{in}}}{\left(K_{1}+K_{12}\right)\left(-1+\kappa_{\mathrm{in}}\right)}$
$\left\{A_{A},B_{A},A_{A},C_{B}\right\}$	$\frac{K_{2}\kappa_{\mathrm{in}}}{1+K_{2}-\kappa_{\mathrm{in}}}$
$\left\{A_{A},B_{A},B_{B},C_{A}\right\}$	$-\frac{K_{2}\left(1+K_{12}\right)\kappa_{\mathrm{in}}}{\left(1+K_{2}\right)\left(K_{12}+\kappa_{\mathrm{in}}\right)}$
$\left\{A_{A},B_{A},B_{B},C_{B}\right\}$	$-\frac{K_{2}\left(1+K_{12}\right)\kappa_{\mathrm{in}}}{\left(1+K_{2}\right)\left(-1+\kappa_{\mathrm{in}}\right)}$
$\left\{A_{A},B_{A},C_{A},C_{B}\right\}$	$\frac{K_{2}\kappa_{\mathrm{in}}}{1+K_{2}}$
$\left\{A_{A},C_{A},B_{B},C_{B}\right\}$	$\frac{\left(1+K_{12}\right)\kappa_{\mathrm{in}}}{\left(1+K_{2}\right)\left(-1+\kappa_{\mathrm{in}}\right)}$
$\left\{A_{A},C_{B},B_{B},C_{B}\right\}$	$-\frac{\left(1+K_{12}\right)\left(1+K_{2}-\kappa_{\mathrm{in}}\right)}{\left(1+K_{2}\right)\left(-1+\kappa_{\mathrm{in}}\right)}$
$\left\{B_{A},C_{A},B_{B},C_{A}\right\}$	$\frac{\left(1+K_{12}\right)\kappa_{\mathrm{in}}}{K_{12}+\kappa_{\mathrm{in}}}$
$\left\{B_{A},C_{A},B_{B},C_{B}\right\}$	$\frac{\left(1+K_{12}\right)\kappa_{\mathrm{in}}}{-1+\kappa_{\mathrm{in}}}$
$\left\{A_{A},C_{A},C_{A},C_{B}\right\}$	$-\frac{\kappa_{\mathrm{in}}}{1+K_{2}}$
$\left\{B_{A},C_{A},C_{A},C_{B}\right\}$	$-\kappa_{\mathrm{in}}$
$\left\{B_{B},C_{B},C_{A},C_{B}\right\}$	$\frac{1-\kappa_{\mathrm{in}}}{1+K_{12}}$

**Table 4:** Kinetico-thermodynamic invariants for a two-step consecutive mechanism, calculated from two different initial conditions: A and C.

Arguments of the F function	Invariant
$\left\{A_{A},B_{A},A_{A},C_{C}\right\}$	$\frac{\left(K_{2}+K_{12}\right)\kappa_{\mathrm{in}}}{K_{1}+K_{12}-\left(1+K_{1}\right)\kappa_{\mathrm{in}}}$
$\left\{A_{A},B_{A},C_{A},C_{C}\right\}$	$\frac{\left(K_{2}+K_{12}\right)\kappa_{\mathrm{in}}}{K_{1}+K_{12}}$
$\left\{A_{A},B_{A},B_{C},C_{C}\right\}$	$-\frac{\left(K_{2}+K_{12}\right)\kappa_{\mathrm{in}}}{1+K_{2}}$
$\left\{A_{A},C_{A},C_{A},C_{C}\right\}$	$-\frac{\left(1+K_{1}\right)\kappa_{\mathrm{in}}}{K_{1}+K_{12}}$
$\left\{A_{A},C_{A},B_{C},C_{C}\right\}$	$\frac{\left(1+K_{1}\right)\kappa_{\mathrm{in}}}{1+K_{2}}$
$\left\{A_{A},B_{C},B_{C},C_{C}\right\}$	$\frac{\left(1+K_{1}\right)\left(-1-K_{2}+\kappa_{\mathrm{in}}\right)}{1+K_{2}}$

**Table 5:** Kinetico-thermodynamic invariants for a two-step consecutive mechanism, calculated from three different initial conditions.

Arguments of the F function	Invariant
$\left\{A_{A},B_{A},B_{B},C_{C}\right\}$	$-\frac{\left(1+K_{12}\right)\left(K_{2}+K_{12}\right)\kappa_{\mathrm{in}}}{\left(1+K_{2}\right)\left(K_{12}+K_{1}\left(-1+\kappa_{\mathrm{in}}\right)+\kappa_{\mathrm{in}}\right)}$
$\left\{A_{A},B_{B},A_{A},C_{C}\right\}$	$\frac{\left(1+K_{1}\right)K_{2}\left(K_{12}+K_{1}\left(1-\kappa_{\mathrm{in}}\right)+\kappa_{\mathrm{in}}\right)}{\left(1+K_{12}\right)\left(K_{1}+K_{12}-\left(1+K_{1}\right)\kappa_{\mathrm{in}}\right)}$
$\left\{A_{A},B_{B},C_{A},C_{C}\right\}$	$\frac{\left(1+K_{1}\right)K_{2}\left(K_{12}+K_{1}\left(1-\kappa_{\mathrm{in}}\right)+\kappa_{\mathrm{in}}\right)}{\left(1+K_{12}\right)\left(K_{1}+K_{12}\right)}$
$\left\{A_{A},B_{B},B_{C},C_{C}\right\}$	$-\frac{\left(1+K_{1}\right)K_{2}\left(K_{12}+K_{1}\left(1-\kappa_{\mathrm{in}}\right)+\kappa_{\mathrm{in}}\right)}{\left(1+K_{2}\right)\left(1+K_{12}\right)}$

**Table 6:** Combinations of perturbed and unperturbed substances in a two-step mechanism.

Unperturbed Substance	Perturbed Substances	Initial Concentrations	Value of δ
A	B, C	(A_o_, B_o_, C_o_) = (A_eq_, B_eq_ – δ, C_eq_ + δ)	δ = B_eq_ – C_eq_
B	A, C	(A_o_, B_o_, C_o_) = (A_eq_ – δ, B_eq_, C_eq_ + δ)	δ = A_eq_ – C_eq_
C	A, B	(A_o_, B_o_, C_o_) = (A_eq_ – δ, B_eq_ + δ, C_eq_)	δ = A_eq_ – B_eq_

**Table 7:** Invariant expressions from the ratio of corrected concentration profiles of A and B: (A_BC_(t) − A_eq_)/(B_AC_(t) − B_eq_). The values of δ_1_, in the first row, correspond to a CPE experiment when A in unperturbed. The values of δ_2_, in the first column, correspond to a CPE experiment when B in unperturbed.

	δ_1_ →	B_eq_	−C_eq_	B_eq_ − C_eq_
δ_2_ ↓				
**A_eq_**		$\frac{\kappa_{\mathrm{in}}}{\kappa_{\mathrm{in}}-1}$	$\frac{-K_{2}\kappa_{\mathrm{in}}}{\kappa_{\mathrm{in}}-1}$	$\frac{-(K_{2}-1)\kappa_{\mathrm{in}}}{\kappa_{\mathrm{in}}-1}$
**−C_eq_**		$\frac{-\kappa_{\mathrm{in}}}{K_{12}\left(\kappa_{\mathrm{in}}-1\right)}$	$\frac{\kappa_{\mathrm{in}}}{K_{1}\left(\kappa_{\mathrm{in}}-1\right)}$	$\frac{(K_{2}-1)\kappa_{\mathrm{in}}}{K_{12}\left(\kappa_{\mathrm{in}}-1\right)}$
**A_eq_ − C_eq_**		$\frac{-\kappa_{\mathrm{in}}}{\left(K_{12}-1\right)\left(\kappa_{\mathrm{in}}-1\right)}$	$\frac{K_{2}\kappa_{\mathrm{in}}}{\left(K_{12}-1\right)\left(\kappa_{\mathrm{in}}-1\right)}$	$\frac{(K_{2}-1)\kappa_{\mathrm{in}}}{\left(K_{12}-1\right)\left(\kappa_{\mathrm{in}}-1\right)}$

**Table 8:** Invariant expressions from the ratio of corrected concentration profiles of A and C: (A_BC_(t) − A_eq_)/(C_AB_(t) − C_eq_). The values of δ_1_, in the first row, correspond to a CPE experiment when A in unperturbed. The values of δ_2_, in the first column, correspond to a CPE experiment when C in unperturbed.

	δ_1_ →	B_eq_	−C_eq_	B_eq_ − C_eq_
δ_2_ ↓				
**A_eq_**		$\frac{k_{1}^{+}}{k_{2}^{+}}=-K_{1}\kappa_{\mathrm{out}}$	$\kappa_{\mathrm{in}}$	$K_{1}\left(K_{2}-1\right)\kappa_{\mathrm{out}}$
**−B_eq_**		$\kappa_{\mathrm{out}}$	$\frac{k_{1}^{-}}{k_{2}^{-}}=-K_{2}\kappa_{\mathrm{out}}$	$-\left(K_{2}-1\right)\kappa_{\mathrm{out}}$
**A_eq_ − B_eq_**		$\frac{K_{1}\kappa_{\mathrm{out}}}{K_{1}-1}$	$\frac{-\kappa_{\mathrm{in}}}{K_{1}-1}$	$-\frac{\left(K_{2}-1\right)\kappa_{\mathrm{in}}}{\left(K_{1}-1\right)K_{2}}$

**Table 9:** Invariant expressions from the ratio of differences of SIC terms and a corrected concentration profile.

**Invariant Expression**	**Values of** **δ**		
	**B_eq_**	**−C_eq_**	**B_eq_ − C_eq_**
$\frac{\chi_{A_{A}}-\chi_{C_{A}}}{A_{\mathrm{BC}}\left(t\right)-A_{\mathrm{eq}}}=-\frac{1+K_{1}+K_{12}}{\delta \left(1+K_{2}\right)}$	$-\frac{{\left(1+K_{1}\left(1+K_{2}\right)\right)}^{2}}{K_{1}\left(1+K_{2}\right)}$	$\frac{{\left(1+K_{1}\left(1+K_{2}\right)\right)}^{2}}{K_{12}\left(1+K_{2}\right)}$	$\frac{{\left(1+K_{1}\left(1+K_{2}\right)\right)}^{2}}{K_{1}\left(-1+K_{2}^{2}\right)}$
$\frac{\chi_{B_{A}}-\chi_{C_{A}}}{A_{\mathrm{BC}}\left(t\right)-A_{\mathrm{eq}}}=-\frac{1+K_{1}+K_{12}}{\delta }$	$-\frac{{\left(1+K_{1}\left(1+K_{2}\right)\right)}^{2}}{K_{1}}$	$\frac{{\left(1+K_{1}\left(1+K_{2}\right)\right)}^{2}}{K_{12}}$	$\frac{{\left(1+K_{1}\left(1+K_{2}\right)\right)}^{2}}{K_{1}\left(-1+K_{2}\right)}$
**Invariant Expression**	**Values of** **δ**		
	**A_eq_**	**−C_eq_**	**A_eq_ − C_eq_**
$\frac{\chi_{B_{A}}-\chi_{C_{B}}}{B_{\mathrm{AC}}\left(t\right)-B_{\mathrm{eq}}}=-\frac{1+K_{1}+K_{12}}{{\delta K}_{1}}$	$-\frac{{\left(1+K_{1}\left(1+K_{2}\right)\right)}^{2}}{K_{1}}$	$\frac{{\left(1+K_{1}\left(1+K_{2}\right)\right)}^{2}}{K_{1}^{2}K_{2}}$	$\frac{{\left(1+K_{1}\left(1+K_{2}\right)\right)}^{2}}{K_{1}\left(-1+K_{1}K_{2}\right)}$
$\frac{\chi_{B_{B}}-\chi_{C_{B}}}{B_{\mathrm{AC}}\left(t\right)-B_{\mathrm{eq}}}=-\frac{1+K_{1}+K_{12}}{{\delta K}_{1}\left(1+K_{12}\right)}$	$-\frac{{\left(1+K_{1}\left(1+K_{2}\right)\right)}^{2}}{K_{1}\left(1+K_{12}\right)}$	$\frac{{\left(1+K_{1}\left(1+K_{2}\right)\right)}^{2}}{K_{1}^{2}K_{2}\left(1+K_{12}\right)}$	$\frac{{\left(1+K_{1}\left(1+K_{2}\right)\right)}^{2}}{K_{1}\left(-1+K_{12}^{2}\right)}$

**Table 10:** Polar two-step submechanisms within linear complex mechanisms.

Linear Complex Mechanism	Polar Two-Step Submechanisms
$\begin{array}{ccccc} & & D & & \\ & & ⇅ & & \\ A & ⇄ & B & ⇄ & C \end{array}$	A⇄B⇄C
	A⇄B⇄D
	C⇄B⇄D
$\begin{array}{ccccc} & & D & & \\ & & ⇅ & & \\ A & ⇄ & B & ⇄ & C\\ & & ⇅ & & \\ & & E & & \end{array}$	A⇄B⇄C
	A⇄B⇄D
	A⇄B⇄E
	C⇄B⇄D
	C⇄B⇄E
	D⇄B⇄E
$\begin{array}{ccccccc} A & ⇄ & B & ⇄ & C & ⇄ & D\\ & & & & ⇅ & & \\ & & & & E & & \end{array}$	D⇄C⇄E
$\begin{array}{ccccccccc} A & ⇄ & B & ⇄ & C & ⇄ & D & ⇄ & E\\ & & & & & & ⇅ & & \\ & & & & & & F & & \end{array}$	E⇄D⇄F
$\begin{array}{ccccccc} & & E & & F & & \\ & & ⇅ & & ⇅ & & \\ A & ⇄ & B & ⇄ & C & ⇄ & D \end{array}$	A⇄B⇄E
	D⇄C⇄F
$\begin{array}{ccccccc} & & & & E & & \\ & & & & ⇅ & & \\ A & ⇄ & B & ⇄ & C & ⇄ & D\\ & & & & ⇅ & & \\ & & & & F & & \end{array}$	E⇄C⇄F
	D⇄C⇄E
	D⇄C⇄F
A⇄B⇄C⇄D	None
$\begin{array}{ccccccccc} A & ⇄ & B & ⇄ & C & ⇄ & D & ⇄ & E\\ & & & & ⇅ & & & & \\ & & & & F & & & & \end{array}$	None

**Table 11:** Invariant expressions obtained from corrected ratios of concentration profiles of single-step substances for some linear complex mechanisms.

Linear Complex Mechanism	Single-Step Substances	Invariant Expression
$A_{1}\begin{array}{c} k_{1}^{+}\\ ⇄\\ k_{1}^{-} \end{array}A_{2}\begin{array}{c} k_{2}^{+}\\ ⇄\\ k_{2}^{-} \end{array}A_{3}$	A_1_, A_3_	$\frac{A_{1\left(2,3\right)}-A_{1,\mathrm{eq}}}{A_{3\left(1,2\right)}-A_{3,\mathrm{eq}}}=-\frac{k_{1}^{-}}{k_{2}^{+}}\frac{\delta_{n}}{\delta_{d}}=\kappa_{\mathrm{out}}\frac{\delta_{1}}{\delta_{2}}$
$A_{1}\begin{array}{c} k_{1}^{+}\\ ⇄\\ k_{1}^{-} \end{array}A_{2}\begin{array}{c} k_{2}^{+}\\ ⇄\\ k_{2}^{-} \end{array}A_{3}\begin{array}{c} k_{3}^{+}\\ ⇄\\ k_{3}^{-} \end{array}A_{4}$	A_1_, A_4_	$\frac{A_{1\left(3,4\right)}-A_{1,\mathrm{eq}}}{A_{4\left(1,2\right)}-A_{4,\mathrm{eq}}}=-\frac{k_{1}^{-}k_{2}^{-}}{k_{2}^{+}k_{3}^{+}}\frac{\delta_{1}}{\delta_{2}}=-\frac{k_{1}^{-}}{k_{3}^{+}}\left(\frac{1}{K_{2}}\right)\frac{\delta_{1}}{\delta_{2}}$
$A_{1}\begin{array}{c} k_{1}^{+}\\ ⇄\\ k_{1}^{-} \end{array}A_{2}\begin{array}{c} k_{2}^{+}\\ ⇄\\ k_{2}^{-} \end{array}A_{3}\begin{array}{c} k_{3}^{+}\\ ⇄\\ k_{3}^{-} \end{array}A_{4}\begin{array}{c} k_{4}^{+}\\ ⇄\\ k_{4}^{-} \end{array}A_{5}$	A_1_, A_5_	$\frac{A_{1\left(4,5\right)}-A_{1,\mathrm{eq}}}{A_{5\left(1,2\right)}-A_{5,\mathrm{eq}}}=-\frac{k_{1}^{-}k_{2}^{-}k_{3}^{-}}{k_{2}^{+}k_{3}^{+}k_{4}^{+}}\frac{\delta_{1}}{\delta_{2}}=-\frac{k_{1}^{-}}{k_{4}^{+}}\left(\frac{1}{K_{2}K_{3}}\right)\frac{\delta_{1}}{\delta_{2}}$
$\begin{array}{ccccccc} A_{1} & \begin{array}{c} k_{1}^{+}\\ ⇄\\ k_{1}^{-} \end{array} & A_{2} & \begin{array}{c} k_{2}^{+}\\ ⇄\\ k_{2}^{-} \end{array} & A_{3} & \begin{array}{c} k_{3}^{+}\\ ⇄\\ k_{3}^{-} \end{array} & A_{4}\\ & & & & \begin{array}{ccc} k^{+} & ⇅ & k^{-} \end{array} & & \\ & & & & A_{5} & & \end{array}$	A_1_, A_4_	$\frac{A_{1\left(3,4\right)}-A_{1,\mathrm{eq}}}{A_{4\left(1,2\right)}-A_{4,\mathrm{eq}}}=-\frac{k_{1}^{-}k_{2}^{-}}{k_{2}^{+}k_{3}^{+}}\frac{\delta_{1}}{\delta_{2}}=-\frac{k_{1}^{-}}{k_{3}^{+}}\left(\frac{1}{K_{2}}\right)\frac{\delta_{1}}{\delta_{2}}$
	A_1_, A_5_	$\frac{A_{1\left(4,5\right)}-A_{1,\mathrm{eq}}}{A_{5\left(1,2\right)}-A_{5,\mathrm{eq}}}=-\frac{k_{1}^{-}k_{2}^{-}}{k_{2}^{+}k^{+}}\frac{\delta_{1}}{\delta_{2}}=-\frac{k_{1}^{-}}{k^{+}}\left(\frac{1}{K_{2}}\right)\frac{\delta_{1}}{\delta_{2}}$
	A_4_, A_5_	$\frac{A_{5\left(3,4\right)}-A_{5,\mathrm{eq}}}{A_{4\left(3,5\right)}-A_{4,\mathrm{eq}}}=-\frac{k^{-}}{k_{3}^{+}}\frac{\delta_{1}}{\delta_{2}}$
$\begin{array}{ccccccccc} A_{1} & \begin{array}{c} k_{1}^{+}\\ ⇄\\ k_{1}^{-} \end{array} & A_{2} & \begin{array}{c} k_{2}^{+}\\ ⇄\\ k_{2}^{-} \end{array} & A_{3} & \begin{array}{c} k_{3}^{+}\\ ⇄\\ k_{3}^{-} \end{array} & A_{4} & \begin{array}{c} k_{4}^{+}\\ ⇄\\ k_{4}^{-} \end{array} & A_{5}\\ & & & & \begin{array}{ccc} k^{-} & ⇅ & k^{+} \end{array} & & & & \\ & & & & A_{6} & & & & \end{array}$	A_1_, A_5_	$\frac{A_{1\left(4,5\right)}-A_{1,\mathrm{eq}}}{A_{5\left(1,2\right)}-A_{5,\mathrm{eq}}}=-\frac{k_{1}^{-}k_{2}^{-}k_{3}^{-}}{k_{2}^{+}k_{3}^{+}k_{4}^{+}}\frac{\delta_{1}}{\delta_{2}}=-\frac{k_{1}^{-}}{k_{4}^{+}}\left(\frac{1}{K_{2}K_{3}}\right)\frac{\delta_{1}}{\delta_{2}}$
	A_1_, A_6_	$\frac{A_{1\left(5,6\right)}-A_{1,\mathrm{eq}}}{A_{6\left(1,2\right)}-A_{6,\mathrm{eq}}}=-\frac{k_{1}^{-}k_{2}^{-}}{k_{2}^{+}k^{+}}\frac{\delta_{1}}{\delta_{2}}=-\frac{k_{1}^{-}}{k^{+}}\left(\frac{1}{K_{2}}\right)\frac{\delta_{1}}{\delta_{2}}$
	A_5_, A_6_	$\frac{A_{6\left(4,5\right)}-A_{6,\mathrm{eq}}}{A_{5\left(3,6\right)}-A_{5,\mathrm{eq}}}=-\frac{k_{3}^{-}k^{+}}{k_{3}^{+}k_{4}^{+}}\frac{\delta_{1}}{\delta_{2}}=-\frac{k^{+}}{k_{4}^{+}}\left(\frac{1}{K_{3}}\right)\frac{\delta_{1}}{\delta_{2}}$
